# Inhibition of the Activating Transcription Factor 6 Branch of Endoplasmic Reticulum Stress Ameliorates Brain Injury after Deep Hypothermic Circulatory Arrest

**DOI:** 10.3390/jcm12030814

**Published:** 2023-01-19

**Authors:** You-Peng Zhang, Qin Yang, Yi-Ai Li, Ming-Huan Yu, Guo-Wei He, Yu-Xiang Zhu, Zhi-Gang Liu, Xiao-Cheng Liu

**Affiliations:** 1 Center for Basic Medical Research, Department of Cardiovascular Surgery, TEDA International Cardiovascular Hospital, Chinese Academy of Medical Sciences, Graduate School of Peking Union Medical College, 61 Third Street, Tianjin 300000, China; 2 Department of Cardiac Surgery, The First Affiliated Hospital, Zhejiang University, Hangzhou 310027, China; 3 School of Pharmacy, Wannan Medical College, Wuhu 241001, China; 4 Department of Surgery, Oregon Health and Science University, Portland, OR 97239, USA

**Keywords:** apoptosis, ATF6, brain injury, deep hypothermic circulatory arrest, endoplasmic reticulum stress

## Abstract

Neurological dysfunction is a common complication of deep hypothermic circulatory arrest (DHCA). Endoplasmic reticulum (ER) stress plays a role in neuronal ischemia-reperfusion injury; however, it is unknown whether it contributes to DHCA-induced brain injury. Here, we aimed to investigate the role of ER stress in a rat DHCA model and cell hypothermic oxygen–glucose deprivation reoxygenation (OGD/R) model. ER stress and apoptosis-related protein expression were identified using Western blot analysis. Cell counting assay-8 and flow cytometry were used to determine cell viability and apoptosis, respectively. Brain injury was evaluated using modified neurological severity scores, whereas brain injury markers were detected through histological examinations and immunoassays. We observed significant ER stress molecule upregulation in the DHCA rat hippocampus and in hypothermic OGD/R PC-12 cells. In vivo and in vitro experiments showed that ER stress or activating transcription factor 6 (ATF6) inhibition alleviated rat DHCA-induced brain injury, increased cell viability, and decreased apoptosis accompanied by C/EBP homologous protein (CHOP). ER stress is involved in DHCA-induced brain injury, and the inhibition of the ATF6 branch of ER stress may ameliorate this injury by inhibiting CHOP-mediated apoptosis. This study establishes a scientific foundation for identifying new therapeutic targets for perioperative brain protection in clinical DHCA.

## 1. Introduction

Aortic arch surgery and the surgical correction of complex congenital heart disease often require deep hypothermic circulatory arrest (DHCA) with cardiopulmonary bypass (CPB) [1]. Since DHCA was first introduced by Dr. Randall Griepp in the 1970s [2], it has been routinely used in aortic arch surgery. Although the overall mortality and morbidity rates after DHCA have declined considerably, neurological dysfunction remains a major complication in patients who have undergone the procedure. Approximately 25% of patients develop transient neurological deficits after DHCA, and the incidence of neuropsychological deficits within 12 weeks after DHCA is as high as 55% [3]. DHCA-related brain impairment is caused by ischemia and hypoxia insult and subsequent reperfusion injury. Systemic inflammatory response during circulatory arrest and apoptotic neuronal death are relevant mechanisms underlying the development of DHCA-related brain injury [4,5,6].

The endoplasmic reticulum (ER) is the primary location for protein processing/modification and calcium ion (Ca^2+^) storage in eukaryotic cells [7]. Blocked protein processing and transportation when cells are exposed to external stimuli can induce the accumulation of misfolded or unfolded proteins in the ER, resulting in ER stress and causing an unfolded protein response (UPR) [8]. ER stress is considered an important factor that contributes to neuronal ischemia/reperfusion (I/R) injury [9]. Moreover, there is evidence showing the activation of inflammatory responses and ER stress in brain tissue in animal models of middle cerebral artery occlusion (MCAO) stroke [10,11,12].

ER transmembrane protein activating transcription factor 6 (ATF6) binds to ER stress elements to amplify the transcription of UPR genes [13]. Evidence suggests that UPR triggers apoptosis if ER stress persists, and the ATF6 pathway of UPR is involved in apoptosis [14]. Micro-ribonucleic acid (miR)-211-5p can ameliorate inflammation and the death of neurons associated with spinal cord injury by targeting and inhibiting the ER stress ATF6 pathway [15]. ATF6 is highly expressed in neurons after intracerebral hematoma, and the level of its downstream protein, the C/EBP homologous protein (CHOP), a transcription factor that promotes apoptosis, is upregulated and peaks at 24 h. Melatonin improves neurological function by inhibiting the ATF6/CHOP/caspase-3 apoptotic pathway [16].

Currently, there are no studies on the role of the ER stress ATF6 pathway in brain injury following DHCA. By establishing a DHCA rat model and hypothermic oxygen-glucose deprivation reoxygenation (OGD/R) cell model, we aimed to determine whether ER stress and its ATF6 pathway are implicated in DHCA-related brain injury, and to provide a scientific basis for the identification of new therapeutic targets for perioperative brain protection in clinical DHCA.

## 2. Materials and Methods

### 2.1. Cell Culture and Drug Intervention

Rat pheochromocytoma PC-12 cells were stored in our laboratory. These cells were cultured in Roswell Park Memorial Institute (RPMI)-1640 (Procell, Wuhan, China) containing 10% fetal bovine serum (Procell, Wuhan, China) in a saturated water environment at 37 °C with 5% carbon dioxide (CO_2_). The culture medium was replaced every alternate day. The PC-12 cells were seeded at a density of 1 × 105 cells/cm^2^.

The cells were plastered for 24 h before treatment with the ER stress inhibitor, 4-phenylbutyrate ([4-PBA], 2 mM) or tauroursodeoxycholate ([TUDCA], 0.2 mM), and the ATF6 inhibitor, Ceapin-A7 (500 nM), for 24 h. ATF6 inhibitory small interfering RNA (siRNA) and a negative control (Jima Genetics, Suzhou, China) were introduced into the cells, as directed by the manufacturer.

### 2.2. Hypothermic OGD/R Experiments

To establish an in vitro DHCA model, a hypoxic chamber (Billups-Rosenberg, San Diego, CA, USA, 95% N_2_/5% CO_2_) was used to simulate an anoxic environment, and cells were cultured in glucose-free RPMI-1640 containing 10% fetal bovine serum at 18 °C. The PC-12 cells were treated with hypothermic OGD/R for 2 h. To rewarm and reoxygenate the cells, they were cultured in a glucose-containing medium in a saturated water environment at 37 °C with 5% CO_2_ for 24 h.

### 2.3. Cell Counting Kit (CCK)-8 Cell Viability Assay and Apoptosis Level Analysis

The cells were cultured in 96-well plates at 16,000 cells per well. Subsequently, 10 μL of CCK-8 solution (Beyotime, Shanghai, China) was added to each well for 4 h after the medium was changed. The cell viability was determined at 450 nm, using a spectrophotometer (Thermo Scientific, Waltham, MA, USA).

### 2.4. Apoptosis Detection

Each group of cells was treated according to the procedure described above. Approximately 0.5–1 × 10^5^ resuspended cells were collected after digestion with 0.25% trypsin, and centrifuged (Eppendorf, Hamburg, Germany) for 5 min at 1000 g. Next, 195 μL of Annexin V-FITC (Beyotime, Shanghai, China) binding solution was added gently to resuspend the cells after discarding the supernatant. Subsequently, 5 μL of binding solution was added, the cells were shaken well, and 10 μL of staining solution was added and mixed gently. The cells were put in an ice bath and then incubated at room temperature (20–25 °C) away from light for 10–20 min. During incubation, the cells were resuspended 2–3 times to improve the staining effect, and apoptosis was detected using flow cytometry (BD, Franklin Lakes, NJ, USA).

### 2.5. Animals and Experimental Protocol

Thirty-two adult male Sprague–Dawley (SD) rats (body mass, 450–520 g; Aochen Experimental Animals, Tianjin, China) were used for experimentation. Feeding management was performed in strict accordance with specific pathogen free-grade rat barrier feeding standards, with two SD rats per cage housed at a room temperature of 24–28 °C with 12 h of alternating light and darkness. The rats had unrestricted access to food and water. The relevant ethical review board approved all the procedures, and the experiments were performed in accordance with institutional and national guidelines and regulations. Thirty-two SD rats were randomly divided into four groups (*n* = 8): the CPB, DHCA, Ceapin-A7 (1 mg/kg/d), and 4-PBA (100 mg/kg/d) groups. The rats in these four groups were injected with saline and inhibitors intraperitoneally for 2 weeks.

### 2.6. DHCA Surgery

Extracorporeal circulation in DHCA was slightly modified, according to a previous experimental operation [10]. A 22G cannula was inserted into the caudal artery, and a homemade multiport catheter was placed into the right jugular vein. The CPB circuit comprised a double-roller pump (Stockert, München, Germany), a homemade blood reservoir, an oxygenator, and a heat exchanger designed for rats (Xi’an Xijing, Xi’an, China). The circulation temperature was controlled using a variable temperature tank (Heater-Cooler System 3T, Stockert, München, Germany). Within 30 min, the core temperature was lowered to 18 °C; subsequently, the extracorporeal circulation was arrested for 45 min. The extracorporeal circulation was then resumed, and the core temperature was slowly rewarmed to 34.5 °C. Next, the extracorporeal circulation was stopped, and ventilation was continued for 1 h under 1.2% isoflurane anesthesia. The rats were extubated and recuperated in an incubator containing oxygen after they resumed spontaneous breathing.

### 2.7. Modified Neurological Severity Scores (mNSS)

The rats were recuperated in an environment containing oxygen for 12 h, and the neurological deficits were scored. The mNSS scores were used to assess the neurological impairments in motor ability, sensory function, reflex activity, and balance performance. If the rats were unable to perform the test or lacked the desired reaction, one point was granted. Higher mNSS scores represented more severe neurological impairments, as follows: 0, no deficit; 1–6, mild deficit; 7–12, moderate deficit; 13–18, severe deficit. Please refer to Table S1 for the specific grading scale. Following neurological deficit scoring, the rats were euthanized.

### 2.8. Histological Study

The entire rat brain was extracted immediately after euthanasia. The brain tissues were fixed in 10% neutral formalin. The brain sections were stained with hematoxylin and eosin (H & E) for histological assessment.

### 2.9. Enzyme-Linked Immunoassay (ELISA)

Blood samples were collected from the rats before euthanasia and were centrifuged for 20 min at 3000 rpm and 4 °C, after which the serum was collected. The serum S100 calcium-binding protein β (S100β) and neuron-specific enolase (NSE) concentrations were determined using ELISA kits (Jianglaibio, Shanghai, China) in accordance with the manufacturer’s instructions.

### 2.10. Western Blotting

Proteins were extracted from rat hippocampal tissue and PC-12 cells using a phenylmethylsulfonyl fluoride-containing lysis buffer (Beyotime, Shanghai, China). Proteins in the gels were transferred to polyvinylidene difluoride membranes to form imprints after effective separation on 10% sodium dodecyl sulfate-polyacrylamide electrophoresis gels (Millipore, Burlington, MA, USA). The following antibodies were used at a concentration of 1:1000: ATF6 (Proteintech, Rosemont, IL, USA); protein kinase RNA-like ER kinase (PERK), phosphorylated-PERK, glyceraldehyde-3-phosphate dehydrogenase, CHOP, caspase-3, activating transcription factor 4 (ATF4), eukaryotic initiation factor-2a (eIF2α), and phosphorylated-eIF2α from Cell Signaling Technology (Boston, MA, USA); and inositol-requiring enzyme 1 (IRE1), phosphorylated-IRE1, x-box binding protein-1 (XBP1), glucose-regulated protein of 78 kDa (GRP78), Bax, Bim, and Bcl-2 from Abcam (Waltham, MA, USA). The primary antibody was incubated with the corresponding secondary antibody (HRP-labeled sheep anti-rabbit antibody; Cell Signaling Technology, Danvers, MA, USA) at room temperature for 1.5 h. The protein bands were analyzed using the Western LightningTM Chemiluminescence Reagent (PerkinElmer, Waltham, MA, USA) and a gel imaging and analysis system (Bio-Rad, Hercules, CA, USA) featuring a camera. The relative intensities of the bands were analyzed using image analysis software (Image J 1.53 K, Wayne Rasband, National Institutes of Health, Bethesda, MD, USA).

### 2.11. Statistical Analysis

All data were presented as mean ± standard deviation (SD). Multiple groups were compared using a one-way analysis of variance, and Tukey’s test was used for post-processing. Statistical analysis was conducted using GraphPad Prism 9.0 (GraphPad, San Diego, CA, USA). Differences were deemed statistically significant when the *p*-value was less than 0.05.

## 3. Results

### 3.1. Changes in ER Stress Protein Expression after DHCA

**Figure 1:**
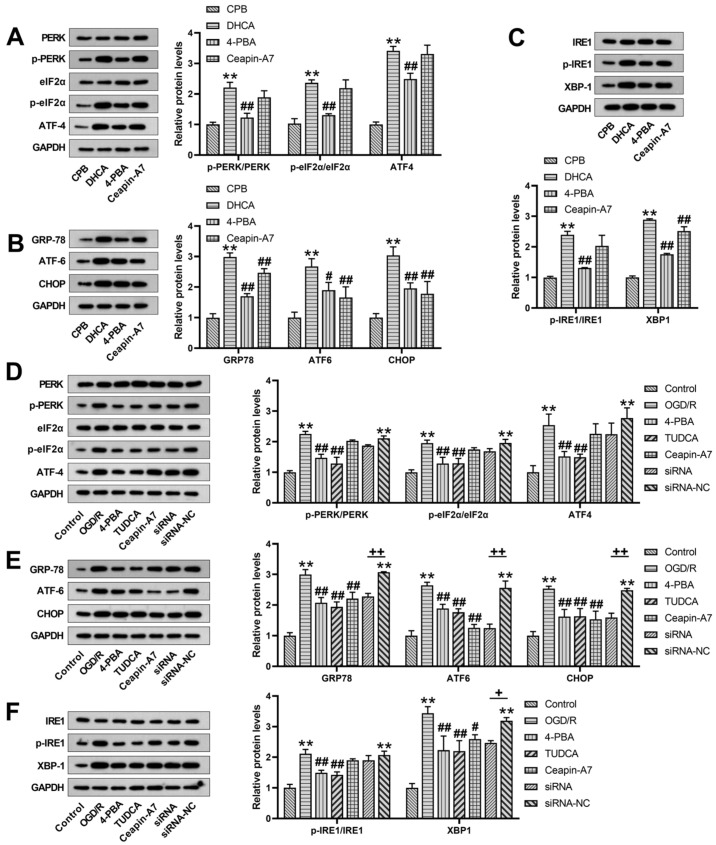
Effectiveness of endoplasmic reticulum (ER) stress inhibitors against deep hypothermic circulatory arrest (DHCA)-induced activation of unfolded protein response (UPR) branches of ER stress in the hippocampus of rats that underwent DHCA (**A**–**C**), and in PC-12 cells exposed to hypothermic oxygen–glucose deprivation reoxygenation (OGD/R) (**D**–**F**). Upregulation/activation of the protein kinase RNA-like ER kinase (PERK)/eukaryotic initiation factor-2a (eIF2α)/activating transcription factor 4 (ATF4), inositol-requiring enzyme 1 (IRE1)/x-box binding protein-1 (XBP1), and activating transcription factor 6 (ATF6)/C/EBP homologous protein (CHOP) UPR signaling pathways was inhibited by the ER stress inhibitor 4-phenylbutyrate (4-PBA) or tauroursodeoxycholate (TUDCA). Specifically, the inhibition of the ATF6 branch of ER stress with Ceapin-A7 treatment downregulated the expression of ATF6 and its downstream molecule, CHOP, without exerting inhibitory effects on the PERK and IRE1 pathways. Data are expressed as the mean ± standard deviation (SD) of at least three independent experiments. Compared with the control or cardiopulmonary bypass (CPB) group, ** *p* < 0.01. Compared with the OGD/R or DHCA group, # *p* < 0.05, ## *p* < 0.01. Compared with the small interfering ribonucleic acid-negative control (siRNA-NC) group, + *p* < 0.05, ++ *p* < 0.01.

To explore whether ER stress occurred during DHCA, PC-12 cells were subjected to hypothermic OGD/R, and the hippocampal tissue extracted from the rats that underwent DHCA was examined for the expression of ER stress molecules. After hypothermic OGD/R and DHCA, the expression of ER stress-related signals in the PC-12 neurons and rat hippocampus, including the expression level of GRP78, ATF4, XBP1, ATF6, and CHOP proteins, were significantly upregulated, and the phosphorylation of PERK, eIF2α, and IRE1 was significantly enhanced, suggesting the induction of ER stress (Figure 1). In rats treated with 4-PBA, and in hypothermic OGD/R cells treated with the ER stress inhibitors, 4-PBA and TUDCA, the expression level of GRP78, a chaperone of the ER, was downregulated, and the PERK, IRE1, and ATF6 pathways of ER stress were inhibited (Figure 1). Ceapin-A7 inhibited the expression of ATF6 in the rat hippocampus, and downregulation of CHOP and GRP78 protein expression was observed (Figure 1A–C). Further experiments targeting the ATF6 branch showed that the treatment of PC-12 cells with the ATF6 inhibitor, Ceapin-A7, or with ATF6-specific siRNA, downregulated the hypothermic OGD/R-induced expressions of GRP78, ATF6, and CHOP (Figure 1D–F).

### 3.2. Inhibition of ER Stress and ATF6-Attenuated DHCA-Induced Brain Injury

**Figure 2:**
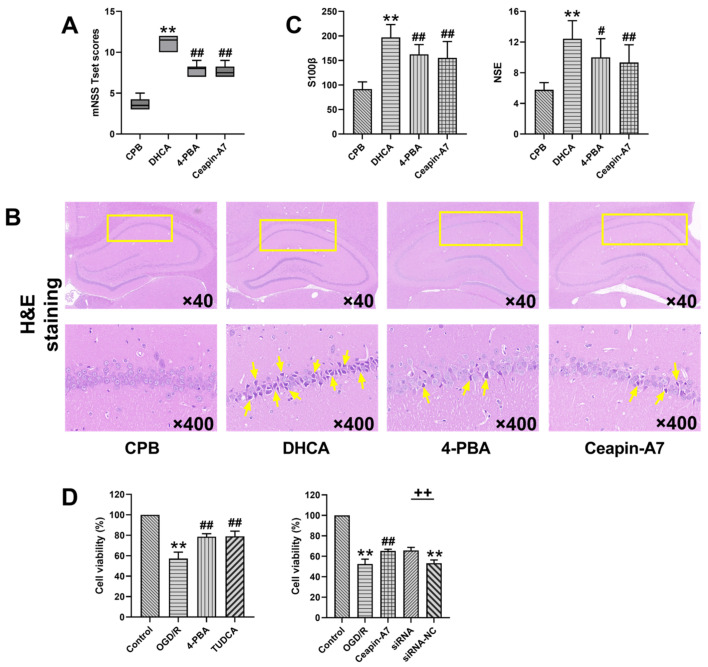
Inhibition of endoplasmic reticulum (ER) stress and inhibition of ATF6 alleviated brain injury caused by deep hypothermic circulatory arrest (DHCA). The modified neurological severity scores (mNSS) in the rats were compared among the cardiopulmonary bypass (CPB) control group, DHCA group, and DHCA group treated with an ER stress inhibitor or activating transcription factor 6 (ATF6) inhibitor (**A**). Brain sections were stained with hematoxylin and eosin. The yellow box indicates the location of the hippocampal CA1 region, and the arrow indicates ischemic neurons (**B**). Comparison of the plasma levels of the brain injury markers, S100 calcium-binding protein β (S100β) and neuron-specific enolase (NSE), in the rats among the CPB control group, DHCA group, and DHCA group treated with 4-PBA or the ATF6 inhibitor (**C**). Comparison of the survival rate after the intervention in the hypothermic oxygen–glucose deprivation reoxygenation (OGD/R)-exposed PC-12 cells treated with or without ER stress inhibitors, an ATF6 inhibitor, or ATF6 small interfering ribonucleic acid (siRNA) (**D**). Data are expressed as the mean ± standard deviation (SD) of at least three independent experiments. Compared with the control or CPB group, ** *p* < 0.01. Compared with the OGD/R or DHCA group, # *p* < 0.05, ## *p* < 0.01. Compared with the siRNA-negative control (NC) group, ++ *p* < 0.01.

The rats in the DHCA group had significantly higher mNSS scores than those in the CPB group, whereas the mNSS scores in the 4-PBA and ATF6 groups were lower than those in the DHCA group, suggesting that the inhibition of ER stress or ATF6 could improve the neurological function of rats after DHCA (Figure 2A). H & E staining showed that a large number of neurons in the hippocampal CA1 region of the rats in the DHCA group exhibited pyknosis, an irregular shape, and a sparse arrangement. Compared to the rats in the DHCA group, those treated with 4-PBA exhibited pyknosis in a small number of neurons in the hippocampal CA1 region. The arrangement of neurons was more compact, and the morphological structure of neurons was normal. In the Ceapin-A7 group, apart from a small number of pyknotic hippocampal CA1 neurons, the remaining cells were tightly arranged, with clear nuclear/ cytoplasmic boundaries and apparent nucleoli (Figure 2B). The ELISA showed that the S100β and NSE levels in the plasma of the rats in the 4-PBA and Ceapin-A7 groups were lower than those in the DHCA group (Figure 2C). The CCK-8 assay showed that hypothermic OGD/R significantly reduced the survival rate of PC-12 cells (control, 100%; hypothermic OGD/R, 57.26%; *p* < 0.01). After treatment with 4-PBA and TUDCA, the cell survival rate increased significantly (control, 100%; 4-PBA, 78.65%; TUDCA, 78.92%; *p* < 0.01). The survival rate of the PC-12 cells was also improved by ATF6 inhibition. Treatment with the ATF6 inhibitor, Ceapin-A7, and ATF6 knockdown increased the survival rate to 65.28% and 65.70%, respectively (Figure 2D). These experiments showed that DHCA caused nerve cell and brain injury, and the inhibition of ER stress and ATF6 attenuated the cell damage and brain injury caused by DHCA.

### 3.3. Inhibition of ER Stress and ATF6-Inhibited Neuronal Apoptosis

**Figure 3:**
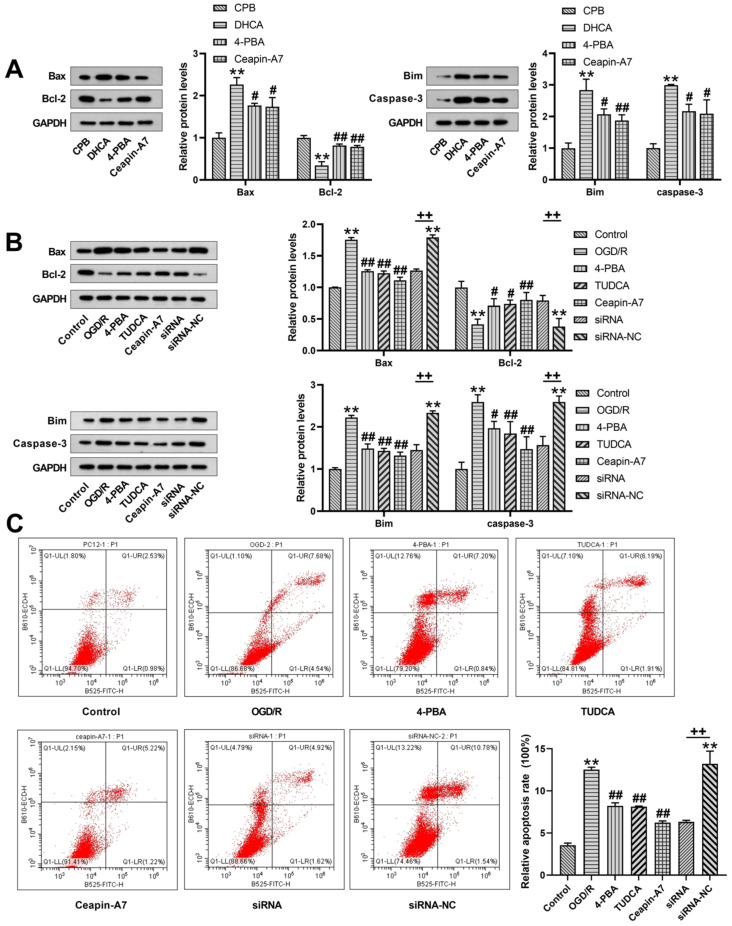
Endoplasmic reticulum (ER) stress inhibitor, 4-phenylbutyrate (4-PBA), and activating transcription factor 6 (ATF6) inhibitor, Ceapin-A7, attenuate deep hypothermic circulatory arrest (DHCA)-induced apoptosis of neuronal cells. Downregulation of the pro-apoptotic proteins, Bax, Bim, and caspase-3, and upregulation of the anti-apoptotic protein, Bcl-2, in the hippocampus of DHCA rats (**A**), and in hypothermic oxygen–glucose deprivation reoxygenation (OGD/R)-exposed PC-12 cells (**B**). Knockdown of ATF-6 in PC-12 cells attenuated hypothermic OGD/R-induced pro-apoptotic protein downregulation and anti-apoptotic protein upregulation (**B**). Inhibition of ER stress or ATF6 reduced PC-12 cell apoptosis after exposure to hypothermic OGD/R (**C**). Data are expressed as the mean ± standard deviation (SD) of at least three independent experiments. Compared with the control or cardiopulmonary bypass (CPB) group, ** *p* < 0.01. Compared with the OGD/R or DHCA group, # *p* < 0.05, ## *p* < 0.01. Compared with the small interfering ribonucleic acid-negative control (siRNA-NC) group, ++ *p* < 0.01.

We verified whether DHCA induces downstream neuronal cell apoptosis, and whether the inhibition of ER stress and ATF6 can ameliorate apoptosis. Western blot experiments revealed that in the hippocampus of DHCA rats and PC-12 neurons exposed to hypothermic OGD/R, the pro-apoptotic proteins, Bax, Bim, and caspase-3 were dramatically upregulated, while the expression of the anti-apoptotic protein, Bcl-2, was inhibited. After the 4-PBA and TUDCA treatment in the cell experiment and 4-PBA treatment in the animal experiment, pro-apoptotic protein expression was inhibited, whereas anti-apoptotic protein expression was upregulated (Figure 3A,B). In the cell experiment, treatment with Ceapin-A7 and siRNA inhibited the pro-apoptotic proteins, Bax, Bim, and caspase-3, and upregulated the anti-apoptotic protein Bcl-2 (Figure 3B). The cell apoptosis assay showed that the level of apoptosis was significantly upregulated after hypothermic OGD/R, while the level of apoptosis in the ER stress inhibition and ATF6 inhibition groups was dramatically lower than that in the hypothermic OGD/R group (Figure 3C). These results suggest that the inhibition of ER stress and ATF6 inhibits DHCA-induced apoptosis.

## 4. Discussion

This study demonstrated, for the first time, that ER stress is activated in the hippocampal tissue of rat brains after DHCA surgery. Additionally, we demonstrated that the inhibition of the ER ATF6 stress pathway can ameliorate DHCA-induced brain injury by inhibiting apoptosis. Therefore, this study provides a scientific basis for identifying new therapeutic targets for perioperative brain protection in clinical DHCA.

S100β and NSE have demonstrated their value in predicting the adverse outcome of the neurological system after cardiopulmonary bypass [17]. In this study, the upregulation of S100β and NSE in the serum of DHCA rats indicated brain injury. The reduced viability of PC-12 cells subjected to hypothermic OGD/R is also evidence of nerve cell injury. Pathological alterations in the hippocampal CA1 region can be detected in the early stage of cerebral ischemia and hypoxia, indicating that the CA1 region is a fragile area, vulnerable to ischemia and hypoxia [18]. In this study, hippocampal tissue damage and neurological dysfunction were observed in DHCA rats, which is consistent with the findings of a previous study [19]. In that same study, the hippocampus of DHCA rats was examined under a transmission electron microscope and dilation and degranulation of the rough ER in the nerve cells was observed, which provided histomorphological evidence for ER disruption.

The Western blot analysis in the present study revealed that the ER stress molecules in the hippocampal tissue were significantly upregulated, which confirmed the presence of ER stress in the brains of rats subjected to DHCA. All three UPR branches of ER stress were activated by DHCA, as shown by the upregulation (increased expression or phosphorylation) of ATF6, PERK, and IRE1, as well as of their downstream signaling molecules, CHOP, eIF2α, ATF4, and XBP1. Additionally, our in vitro experiment on DHCA yielded similar results, where PC-12 cells subjected to hypothermic OGD/R exhibited a significantly upregulated expression of ER stress-related molecules. Taken together, these results suggest that DHCA induces ER stress.

There is evidence that severe ER stress and prolonged activation of ER stress can lead to cell death [20]. Experimental studies of cerebral ischemia have suggested that ER stress is the primary cause of the loss of neurons, glial cells, and endothelial cells during or after cerebral ischemia [21,22,23,24]. In this study, after the inhibition of ER stress in DHCA rats through 4-PBA, we observed amelioration of neurological dysfunction and hippocampal tissue damage, and downregulation of S100β and NSE in the serum after DHCA; this suggested that the inhibition of ER stress ameliorates brain injury after DHCA. Moreover, after DHCA in both the in vivo and in vitro experiments, we observed the upregulation of the pro-apoptotic proteins Bax, Bim, and caspase-3, and the downregulation of the anti-apoptotic protein Bcl-2. The inhibition of ER stress through 4-PBA and TUDCA treatment antagonized DHCA-induced pro-apoptotic proteins upregulation and anti-apoptotic proteins downregulation, in conjunction with a decrease in the apoptosis level and an increase in the cell survival rate, suggesting the involvement of ER stress-mediated apoptosis in DHCA-related brain injury.

Subsequently, we investigated the role of the ATF6 branch of ER stress in DHCA-induced brain injury. The reason we chose ATF6 in this study is that a previous study found that DHCA causes a significant downregulation of rno-circRNA-012043 in rat hippocampal tissue, and miR-211-5p is a predicted target of rno-circRNA-012043 [19]. Taking into consideration the reports showing that miR-211-5p targets ATF6 to reduce neuronal cell death after spinal cord injury [15] and that the inhibition of ATF6 upregulates the ratio of Bcl-2/Bax and downregulates the expression of caspase-3 in rats with focal MCAO [25], we hypothesized that ATF6 mediates neuronal cell apoptosis in DHCA rats. Our results showed that the inhibition of ATF6 ameliorates neurological dysfunction and hippocampal tissue damage after DHCA, and downregulates CHOP expression in both the in vivo and in vitro models of DHCA. The downregulation of the pro-apoptotic transcription factor, CHOP, was accompanied by an enhancement in the expression of the pro-apoptotic proteins Bax, Bim, and caspase-3, and a reduction in the expression of the anti-apoptotic protein Bcl-2 [26,27,28]. These data suggest that CHOP plays a mediating role in ATF6-triggered neuronal cell apoptosis in DHCA. Several studies have reported the role of ATF6-CHOP signaling in apoptosis [29,30,31]. For example, melatonin was found to ameliorate neurological function in an intracerebral hemorrhage model, which may be attributable to the downregulation of the apoptotic proteins associated with ATF6 and downstream CHOP [29]. Apelin-13 can ameliorate neurological function after subarachnoid hemorrhage due to the inhibition of ATF6, which reduced ER stress-induced apoptosis and blood–brain barrier disruption [30]. ATF6 silencing can inhibit Cr (VI)-induced mitochondrial damage, and can reduce the cell apoptosis rate and ER stress level [31]. In a study of the protective effect of melatonin against ischemic neuronal injury, Feng et al. demonstrated PERK/ATF4/CHOP pathway relevance in apoptosis [24]. Considering the upregulation and activation of PERK and ATF4 observed in this study, we assume that the PERK/ATF4/CHOP pathway also participates in DHCA-induced neuronal cell apoptosis; however, this needs to be confirmed by further research.

This study has some limitations. Firstly, in the animal experiments, the neurological impairment and histomorphological changes in the brain tissue were examined only during the perioperative period. Therefore, the long-term effects of DHCA on the neurological function and role of ER stress need to be elucidated in future studies by optimizing the experimental procedure to improve the long-term survival of rats after DHCA. Secondly, the in vitro hypothermic OGD/R experiment could not simulate organ damage, owing to the hemodilution caused by extracorporeal circulation and blood pressure disruption caused by the roller pump; therefore, cell models with a more complex design should be developed to resemble the clinical scenario of DHCA. Thirdly, only a pharmacological intervention was implemented for inhibiting ATF6 in the in vivo experiment. This study could be strengthened by further experiments performed in ATF6 gene-manipulated animals.

## 5. Conclusions

In summary, this study demonstrated that the inhibition of ER stress ameliorated brain injury in rats after DHCA surgery. The downregulation of ATF6 ameliorated DHCA-induced brain injury by inhibiting apoptosis. This study provides a new perspective that could aid in improving brain protection strategies after DHCA cycles. Further investigation of the specific mechanisms underlying the role of ER stress and the ATF6 pathway in DHCA-related brain injury will help in establishing new therapeutic targets for perioperative brain protection in clinical DHCA.

## Data Availability

Data available on request due to privacy restrictions.

## Supplementary Materials

The following supporting information can be downloaded at: https://www.mdpi.com/article/10.3390/jcm12030814/s1, Table S1: Modified neurological severity scores (mNSS).
