# Supplementary material for: Inhibition of the Activating Transcription Factor 6 Branch of Endoplasmic Reticulum Stress Ameliorates Brain Injury after Deep Hypothermic Circulatory Arrest
Source: J Clin Med. 2023 Jan 19;12(3):814. doi: 10.3390/jcm12030814 (PMC9917384; doi:10.3390/jcm12030814)
Supplement: Supplementary file 1 [file jcm-12-00814-s001.zip › Modified neurological severity scores (mNSS).pdf]

Table S1. Modified neurological severity scores (mNSS)

Items	Score
**raising rat by tail (normal=0; maximum=3)**	(3)
flexion of forelimb	1
flexion of hindlimb	1
head moved >10° to vertical axis within 30s	1
placing rat on floor (normal; maximum=3)	(3)
normal walk	0
inability to walk straight	1
circling toward paretic side	2
falls down to paretic side	3
**sensory tests (normal=0; maximum=2)**	(2)
placing test (visual and tactile test)	1
proprioceptive test (deep sensation)	1

**beam balance tests (normal=0; maximum=6)**	(6)
balances with steady posture	0
grasps side of beam	1
hugs beam and 1 limb falls down from beam	2
hugs beam and 2 limbs falls down from beam, or spins on beam(>60s)	3
attempts to balance on beam but falls off(>40s)	4
attempts to balance on beam but falls off(>60s)	5
falls off; no attempt to balance or hang on to beam(<20s)	6
**reflex absence and abnormal movements**	(4)
pinna reflex (head shake when auditory meatus is touched)	1
corneal reflex (eye blink when cornea is lightly touched with cotton)	1
startle reflex (motor response to a brief noise)	1
seizure, myoclonus, myodystony	1
**maximum points**	(18)
